# An Intensive, Active Surveillance Reveals Continuous Invasion and High Diversity of Rhinovirus in Households

**DOI:** 10.1093/infdis/jiy621

**Published:** 2018-12-20

**Authors:** Everlyn Kamau, Clayton O Onyango, Grieven P Otieno, Patience K Kiyuka, Charles N Agoti, Graham F Medley, Patricia A Cane, D James Nokes, Patrick K Munywoki

**Affiliations:** 1 Epidemiology and Demography Department, Kenya Medical Research Institute – Wellcome Trust Research Programme, Kilifi; 2 Centers for Disease Control and Prevention, Nairobi; 3 School of Health and Human Sciences, Pwani University, Kilifi, Kenya; 4 Centre for Mathematical Modelling of Infectious Disease and Department of Global Health and Development, London School of Hygiene and Tropical Medicine, Salisbury; 5 Public Health England, Salisbury; 6 School of Life Sciences and Zeeman Institute for Systems Biology and Infectious Disease Epidemiology Research, University of Warwick, Coventry, United Kingdom

**Keywords:** rhinovirus, transmission, household, developing countries, Kenya

## Abstract

We report on infection patterns in 5 households (78 participants) delineating the natural history of human rhinovirus (HRV). Nasopharyngeal collections were obtained every 3–4 days irrespective of symptoms, over a 6-month period, with molecular screening for HRV and typing by sequencing VP4/VP2 junction. Overall, 311/3468 (8.9%) collections were HRV positive: 256 were classified into 3 species: 104 (40.6%) HRV-A; 14 (5.5%) HRV-B, and 138 (53.9%) HRV-C. Twenty-six known HRV types (13 HRV-A, 3 HRV-B, and 10 HRV-C) were identified (A75, C1, and C35 being most frequent). We observed continuous invasion and temporal clustering of HRV types in households (range 5–13 over 6 months). Intrahousehold transmission was independent of clinical status but influenced by age. Most (89.0%) of HRV infection episodes were limited to <14 days. Individual repeat infections were frequent (range 1–7 over 6 months), decreasing with age, and almost invariably heterotypic, indicative of lasting type-specific immunity and low cross-type protection.

## Teaser

Through an intensive, active surveillance of human rhinovirus (HRV) in households, we observed continuous invasion and high diversity of HRV. Individual repeat infections were frequent, decreasing with age, and almost invariably heterotypic, indicative of lasting type-specific immunity and low cross-type protection.

Human rhinoviruses (HRV) are frequently detected in both children and adults with acute respiratory infections [1, 2], common colds [3], bronchiolitis and pneumonia [4], acute otitis media, and acute wheezing [5]. Although usually mild and self-limiting, HRV infections may exacerbate asthma [6] or other preexisting respiratory illnesses [7], and are sometimes associated with hospitalization with lower respiratory tract infection [8]. In sub-Saharan Africa, HRV prevalence in children <5 years presenting to health care facilities with acute respiratory infections has ranged from 10% to 41% [9–11]. Asymptomatic infections are common and readily detected [12, 13].

HRV is a nonenveloped, single stranded, positive-sense RNA virus of approximately 7200 nucleotides encoding a single polyprotein that is cleaved into 11 proteins [14]. Over 160 types, classified into 3 species (A, B, and C) based on VP1 and VP4/VP2 genes, exist. Rhinovirus A and B types were originally determined by neutralization assays using monospecific antisera and by their specific cellular receptor [15], indicating phenotypic differences between types. Serological assays for routine diagnosis have largely been replaced by molecular methods targeting the 5′ untranslated region gene [16], which permit virus load quantification and have previously been used to describe HRV transmission within households [13, 17–19] and child care centers [20].

HRV is spread by aerosolized droplets or surfaces contaminated with infected respiratory tract secretions, including direct human-to-human contact [21]. To date, no HRV vaccines are available due to antigenic heterogeneity amongst all known strains, lack of data identifying the most commonly circulating strains, or species- or type-specific virulence, and the incomplete understanding of antigenic differences between HRV species [22]. In addition, vaccine development is hindered by lack of estimates of disease burden, and the common assumption that the infections are mild and self-limiting. Nonetheless, HRV is now recognized to lead to more severe disease with relevant impact on patient quality of life and health care-related costs [23].

Transmission is common within families, day care centers and schools, with studies showing several HRV types circulating in a community at the same time [24, 25]. Household and community-based studies provide a natural setting to track infections because of the high frequency of contacts and are a key source of information on viral transmission dynamics [26]. These studies provide estimates of per-infection risk of disease, important insights into epidemiology of respiratory infections, and crucial information for design of public health interventions, for instance, immunization or antiviral prophylaxis [26]. There are no comparative household studies from Africa.

A prospective study, with intensive sampling every 3–4 days, was undertaken to determine the introduction and spread of respiratory syncytial virus in households [27–29]. HRV and other respiratory viruses were detected using a multiplex real-time polymerase chain reaction (PCR) assay [30]. Here, we use the PCR and sequencing data to describe occurrence and frequency of symptomatic and asymptomatic HRV infections and reinfections, and type distribution, in 5 households in a rural coastal setting between December 2009 and June 2010 in Kenya.

## MATERIALS AND METHODS

### Study Area and Procedures

A household-based cohort study was undertaken within the Kilifi Health and Demographic Surveillance System (KHDSS) [31] for 6 months [29]. Briefly, households, eligible if they included an infant and at least 1 older sibling (<13 years), were visited twice a week by trained field assistants who collected nasopharyngeal swab (NPS) samples from all enrolled occupants (regardless of illness) and recorded presence of respiratory symptoms (ie, cough, runny or blocked nose, or difficulty in breathing). Further details of study design and procedures have been previously described [28, 29]. Study protocol was reviewed and approved by Kenya Medical Research Institute Scientific and Ethical Review Unit, Kenya and Coventry Research Ethics Committee, UK. Individual written informed consent was sought from participants (≥18 years) and parents/guardian for the children (<18 years).

### Viral RNA Extraction and Amplification

Viral RNA was extracted using MagNA pure TNA high throughput kit (Roche). NPS samples were screened for respiratory viruses using multiplex real-time reverse-transcription PCR (rRT-PCR) [32]. A sample was determined positive if rRT-PCR cycle threshold (Ct) value was <40.0 [33]. Ct value was assumed to be related to viral load. VP4/VP2 sequencing (approximately 450 bp) was used for species and type assignment. Primers amplifying approximately 549 bp of the VP4/VP2 region (F1: 5′-CCGGCCCCTGAATGYGGCTAA-3′, F2: 5′-ACCRACTACTTTGGGTGTCCGTG-3′; R1: 5′-TCWGG HARYTTCCAMCACCANCC-3′, R2: 5′- ACATRTTYTSN CCAAANAYDCCCAT-3′) were used in a nested 25-μL reaction [34]. PCR products were purified (QIAquick PCR purification Kit, Qiagen) and sequenced on an ABI 3130xl instrument (Applied Biosystems).

### Phylogenetics and HRV Type Assignment

Sequence fragments (forward and reverse-complemented orientations) were compared to form the best possible contig using Sequencher (v5.0, Gene Codes Corp), and multiple alignments generated using MAFFT v6.884b. Maximum likelihood trees were inferred in IQ-TREE [35] and branch support assessed by 1000 bootstrap iterations [36] and SH-like approximate likelihood ratio test [37]. Phylogenetic analyses included VP4/VP2 sequences of HRV prototype strains (http://www.picornaviridae.com/sequences/sequences.htm). Type assignment was based on sequence similarity to prototype strains and pairwise genetic distance: phylogenetic clustering (bootstrap value >80%) and nucleotide divergence thresholds previously proposed by McIntyre et al (10.5%, 9.5%, and 10.5% for HRV-A, -B, and -C, respectively) [15]. Distributions of pairwise distances were computed to check for clear thresholds for defining intertype and intratype divergence values.

### Descriptive and Statistical Methods

An infection episode (either individual or household) was defined as the period when the same HRV type was detected with no more than 14 days between any 2 positive samples. This was supported by previous observations that rhinovirus shedding lasts on average 10–14 days (illness resolves within 1–2 weeks) [38, 39]. HRV-positive untyped samples were assumed to be of the same type as samples of the episode within which they were sandwiched. Household outbreaks were defined as the occurrence of more than 1 individual infection episodes with the same HRV type in a household and no more than 14 days between the infection episodes. A primary or index case was the first person with PCR-confirmed HRV within the same household outbreak while secondary case(s) were the rest of the individual infection episodes in the same household outbreak. Durations of infection episodes were crudely estimated as the date of last positive sample minus date of first positive plus 1. An individual or household infection episode was defined as symptomatic if at least 1 sample within the episode coincided with presence of 1 or more respiratory symptoms.

Categorical variables were summarized as frequencies and their corresponding percentage distributions. Summaries for continuous variables were reported as mean and standard deviation (SD), and if found to have a skewed distribution were reported as median and interquartile range (IQR). Differences in proportions between groups were determined using test of proportions. Test for linear trend was used to investigate the trend in proportion of symptomatic cases with increasing age. We used logistic regression to estimate odds ratios (ORs) for the association between demographic and clinical characteristics and HRV infection status and generalized estimating equations with exchangeable covariance structure to account for repeated entries of the same individual. Kaplan-Meier functions were used to estimate survival rates for HRV reinfections, and log-rank test for between-group comparisons. Cox proportional hazard regression was used to obtain hazard ratios (HR) with 95% confidence intervals (CI) for incidence rate comparison. All statistical analyses were done using STATA 13.1 (Statacorp), and effects were considered significant for *P* values ≤ .05. Epiplots depicting distribution of HRV types within households were plotted in R v3.2.1. The replication datasets, do files, and R scripts are available on the Dataverse site (https://doi.org/10.7910/DVN/NDJFNZ) [40]. The Genbank sequence accession numbers are KX831136–KX831389.

## RESULTS

Five households, designated 5, 19, 34, 40, and 51 and consisting of 78 individuals in total, were selected to represent the breadth of household sizes in the larger cohort for detailed analysis on HRV infections. The minimum and maximum distance between the five households was 0.3 and 1.8 kilometers, respectively. The number of occupants (and median age in years) was 37 (11.4), 14 (13.0), 7 (7.4), 5 (6.1), and 15 (9.2) for households 5, 19, 34, 40, and 51, respectively. Overall, 11% of participants were infants (<12 months of age), 23% were 1 to ≤5 years, 17% were 6 to ≤10 years, 17% were 11 to ≤18 years, and 32% were >18 years.

### HRV Detections, Species, and Types

**Figure 1.**
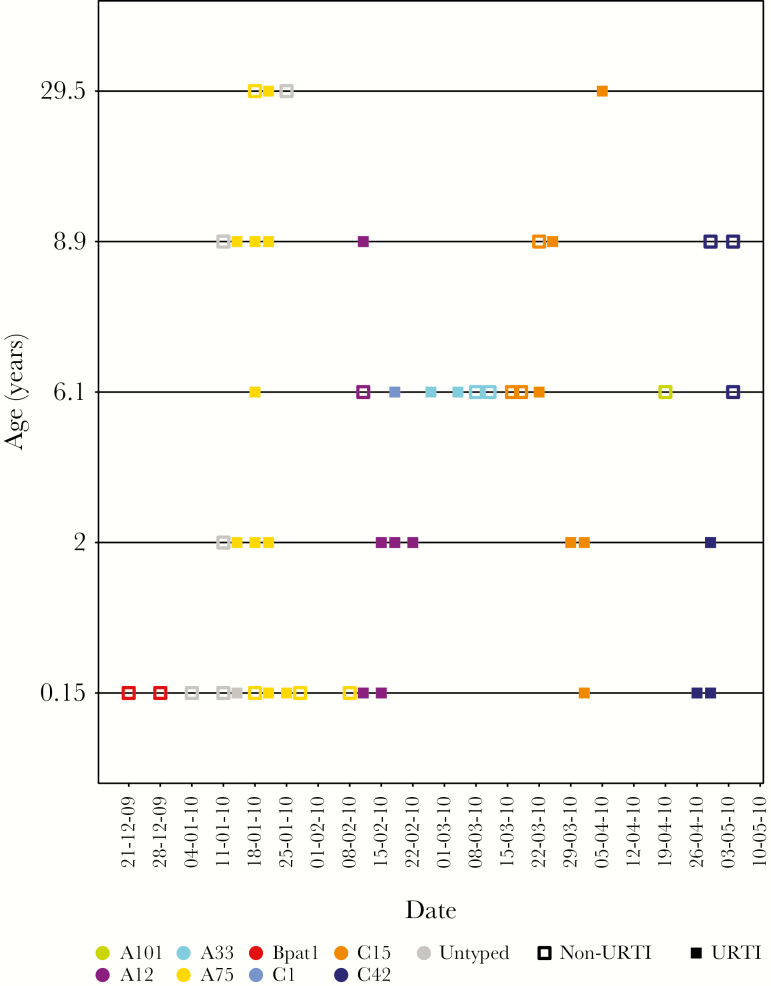
Distribution of human rhinovirus-positive samples collected in household 40, 1 of the 5 households in coastal Kenya, by individual and coded by type (color) and symptom status (filled markers, symptomatic; empty markers, asymptomatic) at time of sample collection. As shown, age increases from the infant at the bottom to the oldest member of the household. The other households are shown in the Supplementary Figure 2. Abbreviation: URTI, upper respiratory tract infection.

**Figure 2.**
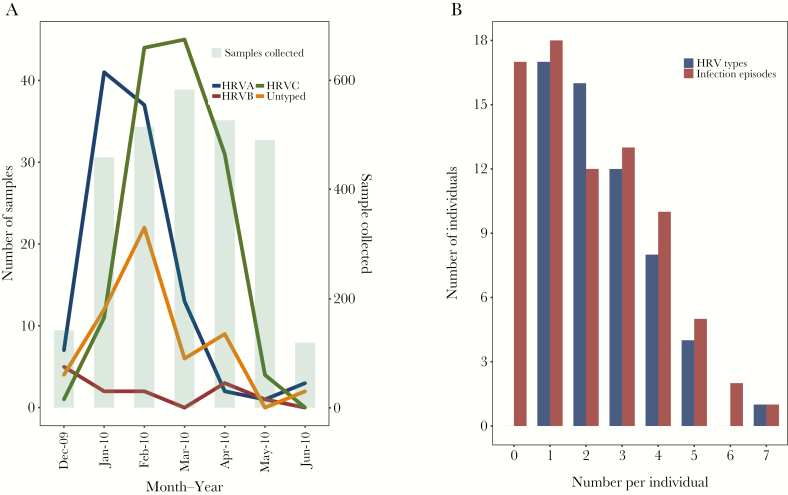
For a set of 5 households from coastal Kenya sampled between December 2009 to June 2010, (*A*) monthly occurrence of human rhinovirus, by species, with numbers of samples collected per month, (*B*) distribution of number of infection episodes and HRV types, per individual.

The total number of NPS collections from the 5 households was 2836 and of these 313 (11%) were HRV positive. The VP4/VP2 region (approximately 420 bases) was sequenced for 256 rRT-PCR positive samples (82.3%); the remaining samples either failed to amplify with the VP4/VP2 specific primers (55) or were identified as non-HRV enteroviruses (2). Of the 55 untyped samples, 27 (49.1%) occurred within individual infection episodes and therefore were assumed to be of the same type as samples of the corresponding episode within which they were sandwiched. Overall, 104 (40.6%), 14 (5.5%) and 138 (53.9%) were HRV-A, -B, and -C, respectively. Additionally, 13 HRV-A, 3 HRV-B, and 10 HRV-C defined types were identified (total of 26) and are listed by household in Table 1. The distribution of HRV-positive samples over the study period are shown in Figure 1 for household 40. Similar plots for other households are in the Supplementary material. Figure 1 demonstrates 3 common features amongst all households: (1) multiple invasions, almost always of different types, with (2) each household outbreak lasting a short period of days to a few weeks, and (3) individuals were multiply infected with different types. Species A and C were detected in all 5 households, whereas HRV-B was detected in 4 (Table 1). C35 (n = 44) and C1 (n = 33) were the most commonly detected HRV-C types; A75 (n = 27) was the most frequent HRV-A type; and Bpat1 (n = 10) the most common HRV-B type. The proportion of HRV-positive samples was significantly different (*P* value < .001) across age groups: <1 year (n = 81; 28.9%), 1–5 years (n = 102; 14.7%), 6–10 years (n = 67; 13%), 11–18 years (n = 32; 7.2%), and >18 years (n = 31; 4.4%). Figure 2A shows the temporal distribution by species, with peak occurrence January–February for HRV-A, February–March for HRV-C, and no clear pattern for HRV-B. Supplementary Figure 1 shows the distribution of various age groups in relation to prevalence of rhinovirus species.

**Table 1.** Surveillance for Human Rhinovirus Types in 5 Households in Coastal Kenya 2009–2010

HRV type	HH 5 (37)	HH 19 (14)	HH 34 (7)	HH 40 (5)	HH 51 (15)
**A75**	+	ND	ND	+	ND
**A60**	+	+	ND	ND	ND
**A12**	+	ND	ND	+	ND
**A7**	+	ND	ND	ND	ND
**A46**	+	+	ND	ND	ND
**A65**	ND	ND	ND	ND	+
**A62**	+	ND	ND	ND	ND
**A66**	ND	+	+	ND	ND
**A43**	ND	ND	+	ND	+
**A33**	ND	ND	ND	+	ND
**A98**	ND	ND	ND	ND	+
**A2**	ND	ND	ND	ND	+
**A101**	ND	ND	+	+	ND
**B3**	+	ND	ND	ND	ND
**B37**	ND	+	ND	ND	ND
**Bpat1**	ND	+	+	+	ND
**C1**	+	ND	ND	+	+
**C15**	+	+	ND	+	ND
**C53**	+	ND	ND	ND	ND
**C5**	+	ND	+	ND	ND
**Cpat19**	+	ND	ND	ND	ND
**C35**	+	+	ND	ND	+
**C42**	ND	+	ND	+	ND
**C43**	ND	ND	ND	ND	+
**Cpat16**	ND	ND	ND	ND	+
**C52**	ND	ND	ND	ND	+
**Total**	13	8	5	8	9

Abbreviations: +, detected; HH, household; ND, not detected.

Number of occupants for each household is indicated in the first row.

### HRV Episode Distribution

Overall, 61/78 (78.2%) individuals experienced 1 or more HRV episode; typing failed in 3. Eighteen (23.7%) had a single episode over the 6-month observation period, while 43 (55.1%) had >1, up to a maximum of 7, infection episodes (Figure 2B). Cumulatively, there were 163 individual infection episodes (<14 days between any 2 positive samples) (Supplementary Figure 2), resulting in a mean of 2.24 (SD, 1.29) episodes per person. The distribution of number of types per individual followed a similar pattern to the number of episodes (Figure 2B), with up to a maximum of 7 types, indicating very few instances of reinfection with the same type. There were 56 household infection episodes, involving 1 or more individuals, where 23, 7, and 26 episodes were HRV-A, -B, and -C, respectively, ranging from 8 in households 34 and 40 to 18 in household 5 (Supplementary Figure 2). Of the 56 household episodes, 26 (46%) led to secondary cases (household outbreaks), ranging from 3 in household 51 to 7 in households 5 and 19. The number of HRV types per household ranged from 5 in household 34 to 13 in household 5 (Supplementary Figure 1; Table 1). The duration of individual episodes ranged from 1 to 36 days (median 1 day; Supplementary Figure 2), while duration of household episodes ranged from 1 day to 40 days (median 8 days; Supplementary Figure 3).

### Clinical Features and Transmission

**Figure 3.**
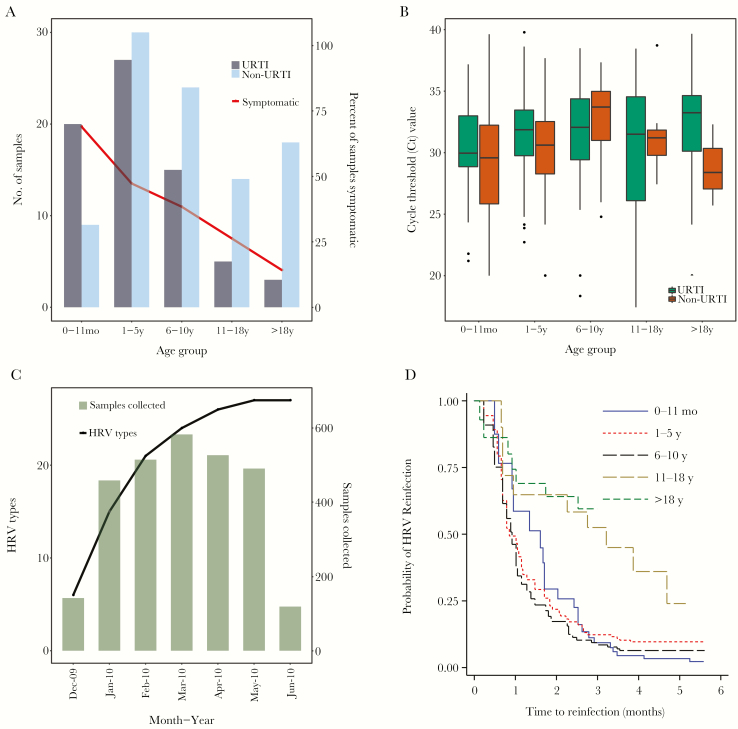
Surveillance in 5 households in 2009–2010 in coastal Kenya. (*A*) Human rhinovirus (HRV)-positive samples by age groups and by symptomatic status, with proportions per group shown as percentage. (*B*) box plots of real-time reverse-transcription polymerase chain reaction cycle threshold (Ct) values across the various age groups, by symptom status. (*C*) cumulative number of the different HRV types observed over the study period in all the 5 households (line), comparing with monthly sample collection values (bars). (*D*) Kaplan-Meier plot showing survival estimates of time to HRV reinfection, stratified into 5 age groups. Abbreviations: URTI, upper respiratory tract infection

Amongst the 163 HRV individual episodes, 94 (58%) were asymptomatic and 69 (42%) were symptomatic: 26% had nasal discharge or blockage, 40.5% had cough, and 0.6% had difficulty breathing. The proportion of HRV-positive samples concurrent with presence of symptoms declined with increasing age (Figure 3A). The number of HRV-positive samples derived from asymptomatic individuals was 63.9% (200/313). Association between infecting rhinovirus species and presence or absence of respiratory symptoms, adjusted for age, was not significant for HRV-B (OR = 0.30; 95% CI, 0.07–1.28; *P* value = .104) or HRV-C (OR = 0.63; 95% CI, 0.36–1.08; *P* value = .092), using HRV-A as the reference group. Excluding samples positive for other viruses tested, specimens collected while symptomatic had double the odds of having a HRV infection compared to asymptomatic ones (OR = 2.01; 95% CI; 1.42–2.85). Of the 56 household episodes, 24 (42.9%) were asymptomatic and 32 (57.1%) were symptomatic. The proportion of symptomatic individual episodes declined with increasing age: infants (66.7%), 1–5 years (48.2%), 6–10 years (38.4%), 11–18 years (26.3%) and >18 years (14.3%) (*Χ*^2^ = 16.7; *P* value < .001).

A linear regression fitted on Ct and age, and using a piecewise linear function for age, showed a statistically significant positive relationship for children <11 years (*β* = 0.22; 95% CI, 0.09–0.35; *P* value = .001) and a negative, not statistically significant, relationship for older children and adults ≥11 years (*β* = −0.044; 95% CI, −0.1 to 0.1; *P* value = .111). This was unaffected by adjusting for reported symptoms: <11 years (*β* = 0.198; 95% CI, 0.06–0.33; *P* value = .004) and ≥11 years (*β* = −0.047; 95% CI, −0.1 to 0.008; *P* value = .092). Overall, age and symptomatic status explained little of the variation in amount of virus shed in both adjusted and unadjusted models (*R*^2^ = 0.03). Slight differences between median Ct value of symptomatic and asymptomatic cases were observed across age categories (Figure 3B). Association between symptomatic status of the index cases and within household spread was not statistically significant (unadjusted OR = 1.14; 95% CI, 0.41–3.14; *P* value = .801) and did not change after adjusting for age in years (adjusted OR = 1.02; 95% CI, 0.35–2.96; *P* value = .966). There was no statistically significant association between viral shedding (high Ct ≤30 versus low Ct >30, using low as reference category) of the index case and that of secondary cases (with the same type) in the household (OR = 1.86; 95% CI, 0.67–5.17; *P* value = .233).

### HRV Infection and Reinfection Dynamics

Overall, 11/26 types were observed in multiple households. The cumulative number of unique types increased rapidly initially then less so with time (Figure 3C). Circulation of any particular type was restricted temporally, and heterologous types, rather than long-term persistence of a single type, caused rhinovirus reinfections. The slope from linear regression analysis of household size (population density) and HRV type diversity was 0.204 (95% CI, 0.02–0.38; *P* value = .036). Four individuals had same-type reinfections Supplementary Figure 2 a 6.6 year old in household 5 with type A75; a 2.3 year old in household 34 with type A43; a 4.6 year old in household 34 with type C5; and a 1.5 year old in household 51 with type C43. At household level, recurrence of the same type (C5 and C15 in household 5; A54, C43 and Cpat16 in household 51) was observed after long (>1 month) intervening periods. Notably, there was a pronounced symptomatic spread of C43 in household 51 after a period of presumed viral absence, (6 weeks after the first 2 asymptomatic introductions).

Children younger than 10 years were more at risk of “all HRV” reinfection(s) compared to older individuals. Using infants (0–11 months) as the reference category, older children (11–18 years) and adults (>18 years) had a reduced risk of rhinovirus reinfection with HRs of 0.34 (95% CI, 0.14–0.80; *P* value = .013) and 0.17 (95% CI, 0.06–0.48; *P* value = .001), respectively (Figure 3D). However, compared to infants, the risk of reinfection was not significantly lower for children 1–5 years (HR = 0.79; 95% CI, 0.53–1.17; *P* value = .242) or for those 6–10 years (HR = 0.90; 95% CI, 0.57–1.42; *P* value = .65) (Figure 3D).

### HRV Type Sequence Diversity

**Figure 4.**
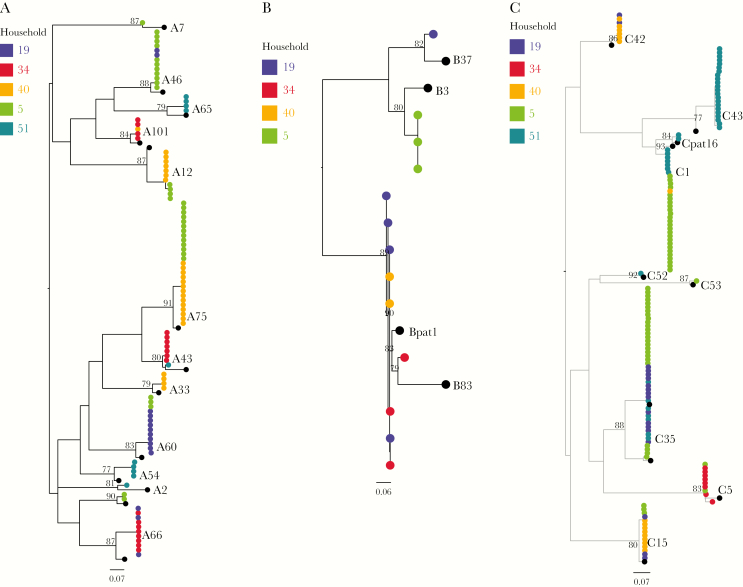
Human rhinovirus (HRV) VP4/VP2 phylogenies from coastal Kenya 2009–2010, showing relationships of sequences between the 5 households: (*A*) HRV-A, (*B*) HRV-B, and (*C*) HRV-C. Tips are colored by household of origin and sequences of prototype strains are colored in black. Branch lengths were assigned with SH-aLRT and UFBoot support values. Scale bar indicates nucleotide substitutions/site.

Strains of types A101, A75, A66, A46, C42, C1, C15, and C35, observed in more than 1 household, each had close sequence similarity (>90%) and grouped into monophyletic clusters irrespective of household of origin (Figure 4), suggesting a single virus variant circulated in the community. There were distinct clusters (>80% bootstrap) for HRV types A12, A43, and C1 between households denoting diversification following transmission within the community. Two HRV types were unusually different genetically within households: C43 in household 51 and C5 in household 34. Two C5 variants in household 34, detected >14 days apart, had 16 nucleotide and 12 amino acid differences.

Comparing the household sequences with those collected during the same period from Kilifi County Hospital (KCH), a facility that serves a larger catchment area of Kilifi County, HRV-A and -C viruses from household and KCH grouped in the same monophyletic clusters (Supplementary Figure 4), confirming existence of these strains within the wider Kilifi County. Average within-species pairwise distances for HRV-A, -B, and -C were 0.182, 0.101, and 0.211, respectively. Supplementary Table 1 shows *p* distances between household HRV sequences and their corresponding prototype strains. A66, A65, A2, B3, and B37 strains varied considerably from their reference strains: their *p* distances failed to conform to previously proposed minimum VP4/VP2 type assignment thresholds of 10.5% and 9.5% for HRV-A and HRV-B, respectively, despite strong bootstrap-supported (>90%) monophyletic clustering. Notwithstanding, these viruses remained classified as A66, A65, A2, B3, and B37.

## DISCUSSION

The household setting represents an epidemiologic niche in which conditions facilitate respiratory disease transmission due to close and frequent personal contacts. Here, 5 households (78 individuals), repeated follow-up, sampling twice weekly, and molecular typing allowed detection of recurrent or serial HRV infections from an otherwise healthy population, providing insights into natural infection history and transmission dynamics. Diverse types were in circulation. Individuals and households were infected up to 8 and 13 times, respectively. The vast majority of reinfections were with heterologous types, as previously observed [12, 41]. There was little genetic diversity among infected individuals both within and between households. With time, the number of new types saturated in the households, although other types circulated in the wider community as identified by contemporaneous local hospital surveillance. This showcases continuous exposure to a substantial but temporally restricted subset of invariant types in a local household population, where reinfection is constrained by strong homotypic but not heterotypic immunity. Findings from a recently published day care center study, using similar study design (longitudinal study collecting samples from symptomatic and asymptomatic individuals and positives genotyped), show a high proportion positive in symptomless individuals, high diversity of types in circulation, and repeat infections are often heterotypic [20].

These observations imply that duration of immunity to heterologous types is short and at most of the order of weeks, and that of homologous reinfection of the order of months. However, full duration of immunity to same-type reinfection could not be estimated here. Further studies of a longer period would shed more light on type-specific immunity. A decrease in reinfection rates with increasing age suggests a broader cross-type immunity in individuals with more HRV exposure. The first infections observed during the study might have occurred rapidly due to faded immunity over the preceding year to the same types. In this study period, transition from A to C, with B being relatively rare, suggests a feasible “exclusion” mechanism.

Most infection episodes were asymptomatic and absence of symptoms was not associated with lower infectivity; a significant proportion of asymptomatic infections contributed to transmission. This is important when estimating the rate of community HRV infection as it leads to underestimation if sampling is based on presence of symptoms. While some types were more common, there was no evidence of species or types associated with presence of symptoms. HRV infections were more common in younger relative to older individuals, with duration, virus shedding, reinfection rate, and proportion symptomatic, decreasing with increasing age, consistent with the notion that a longer history of exposure and disease in adults compared to children accounts for the diversity of clinical presentation and patterns of viral shedding [42, 43]. A wide range of ages and substantial proportion of the household were involved in household outbreaks.

The observation of saturation of numbers of new types infecting households with increasing time could have multiple reasons including: (1) increasing type-specific immunity amongst the community of households, reducing the risk of continued spread of identified types; (2) near exhaustion of a finite set of types circulating in the community with which to infect households; or (3) a reflection of changes in the numbers of samples collected. The latter is less plausible. In spite of the significant linear relationship between household size (population density) and HRV types detected, caution should be taken in over interpretation as we are dealing with only 5 data points (households).

Rhinoviruses with *p* distances above minimum type assignment thresholds indicate considerable nucleotide divergence from the closest prototype strains. Our findings extend previous observations, using serologic or genotypic analysis [19, 44, 45], of differences in frequency of HRV occurrence. The strengths of our study included selection of participants and regularly scheduled prospective sampling independent of health history and irrespective of respiratory symptoms, giving an unbiased view on HRV presence in a rural community. Nonetheless, the study has limitations and assumptions: (1) our findings were restricted to the VP4/VP2 region whereas full genomes would have been useful for more detailed epidemiologic (who infects whom) and evolutionary analyses; (2) the small number of households studied might not be fully representative of the entire household cohort or generalizable to other settings; (3) the short duration of follow-up limits insights into duration of immunity; and (4) we assumed time-independent HRV exposure when estimating infection rates. These notwithstanding, the temporal patterns of HRV acquisition, and identification of similar HRV types in households and pediatric hospital admissions, suggest a generalizability of our results to a larger population. This study highlights the natural history of rhinovirus infections within a household setting, revealing patterns of viral shedding, and rates of infection and reinfection in relation to type diversity, age, and clinical symptoms.

## Supplementary Data

Supplementary materials are available at *The Journal of Infectious Diseases* online. Consisting of data provided by the authors to benefit the reader, the posted materials are not copyedited and are the sole responsibility of the authors, so questions or comments should be addressed to the corresponding author.

Supplementary Figure S1

Supplementary Figure S2

Supplementary Figure S3

Supplementary Figure S4

Supplementary Figure S5

Supplementary Material

## Notes

***Acknowledgments.*** We thank the study participants and members of the Virus Epidemiology and Control Research Group in Kilifi who were involved at various stages of this study, including sample and data collection and laboratory screening. The study is published with permission of the director of Kenya Medical Research Institute.

***Financial support.*** This work was supported by the Wellcome Trust (grant numbers 090853, 102975, and 203077).

***Potential conflicts of interest.*** All authors: No reported conflicts of interest. All authors have submitted the ICMJE Form for Disclosure of Potential Conflicts of Interest. Conflicts that the editors consider relevant to the content of the manuscript have been disclosed.
