# Supplementary figures and images for: An Intensive, Active Surveillance Reveals Continuous Invasion and High Diversity of Rhinovirus in Households
Source: J Infect Dis. 2018 Dec 20;219(7):1049–57. doi: 10.1093/infdis/jiy621 (PMC6420174; doi:10.1093/infdis/jiy621)

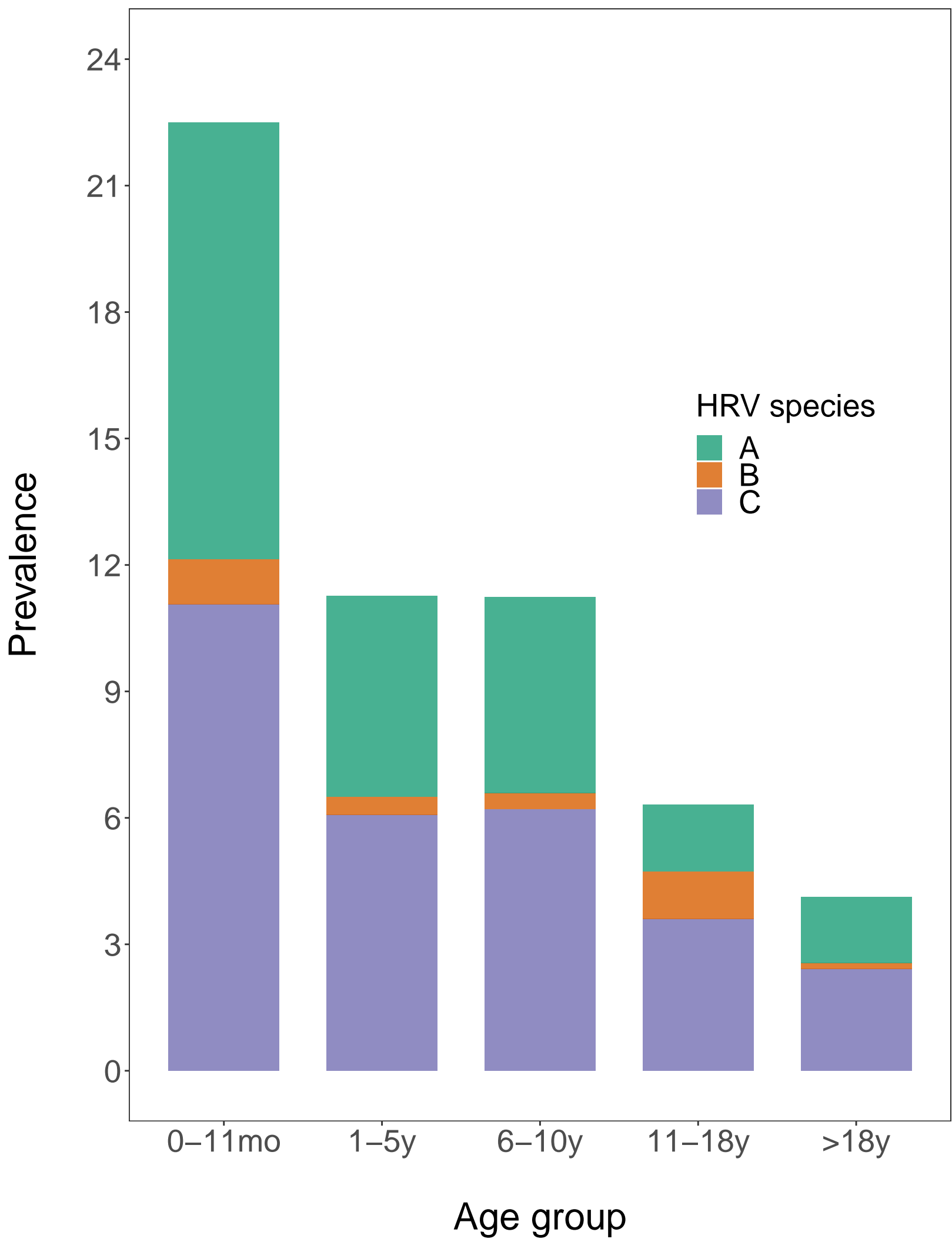

Supplement: Supplementary Figure S1 [file jiy621_suppl_supplementary_figure-s1.pdf]

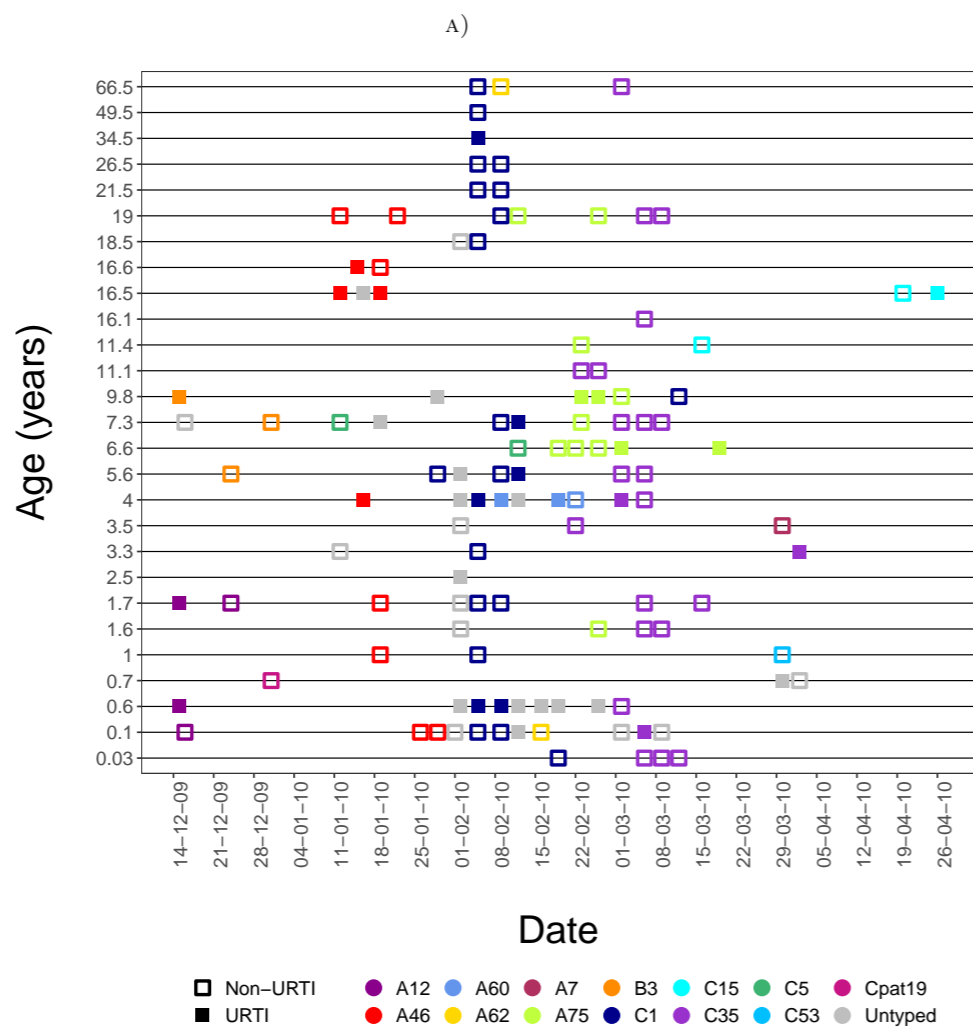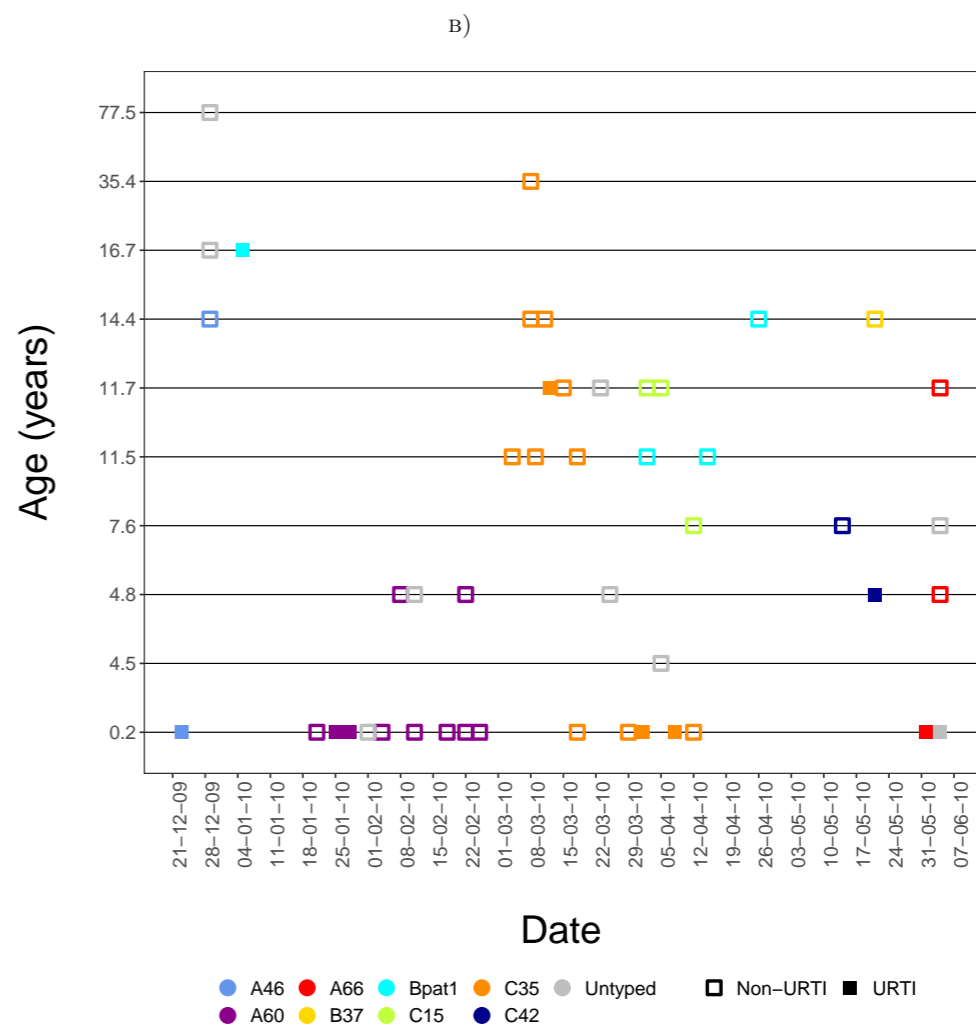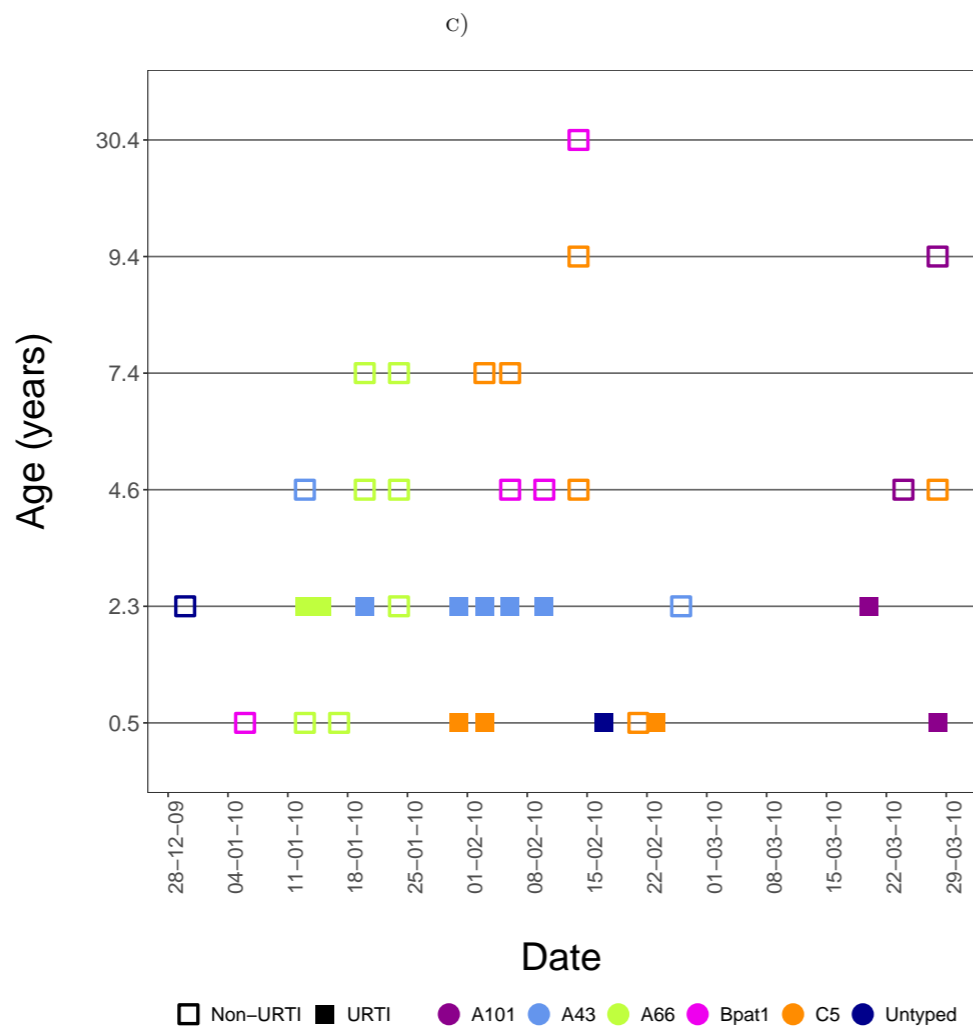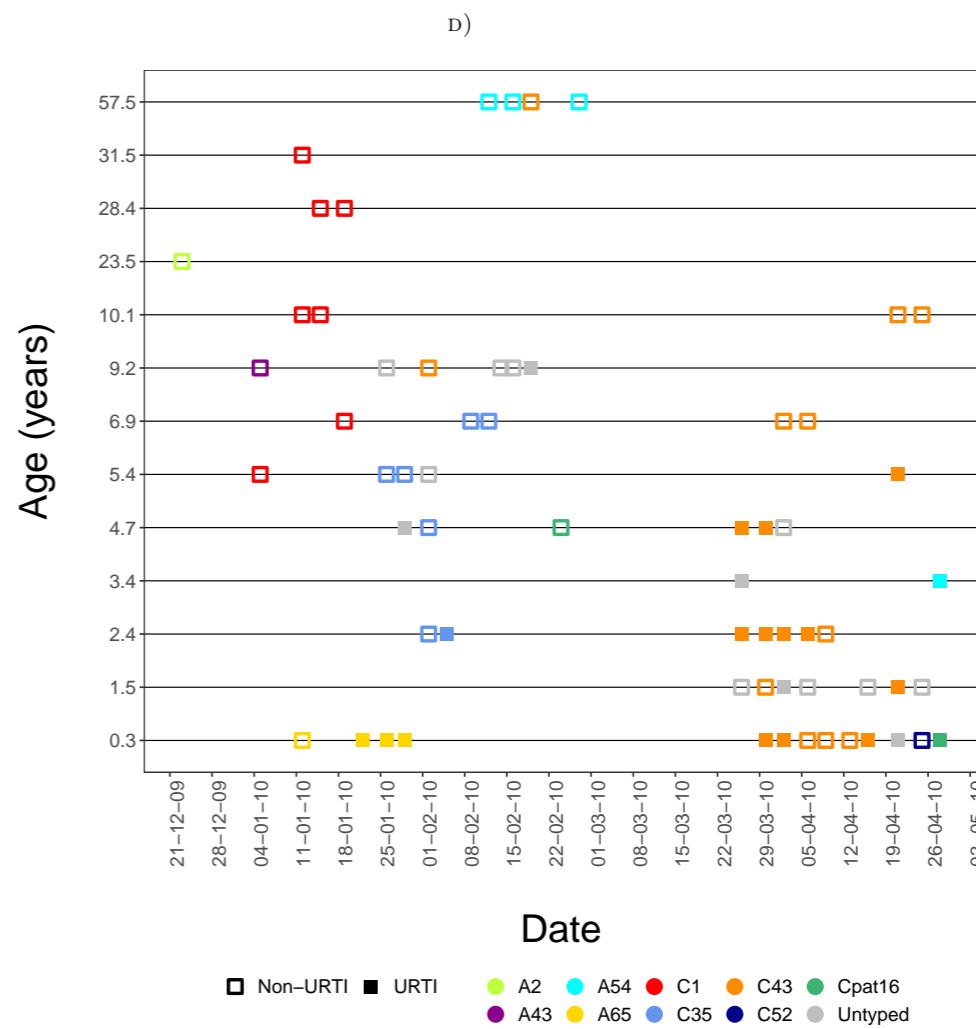

Supplement: Supplementary Figure S2 [file jiy621_suppl_supplementary_figure-s2.pdf]

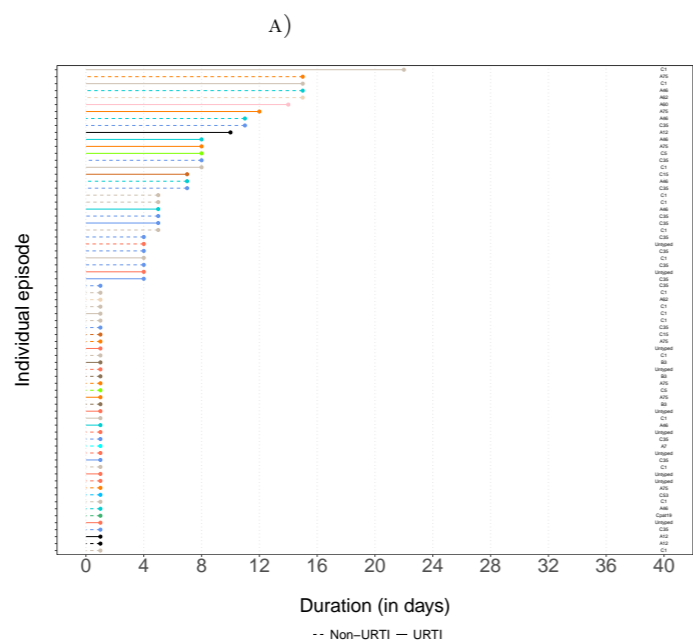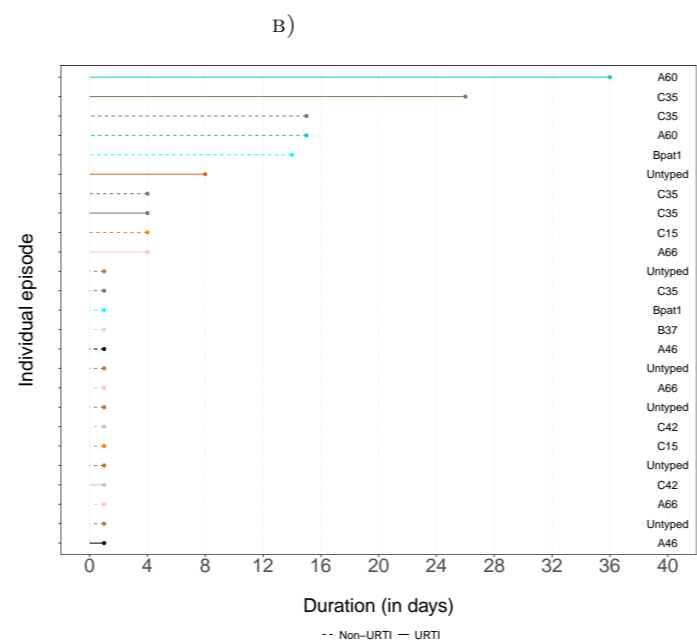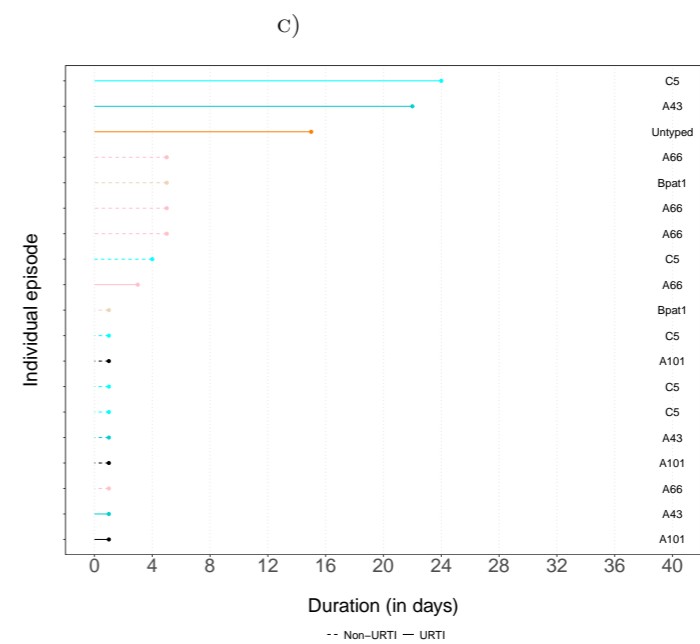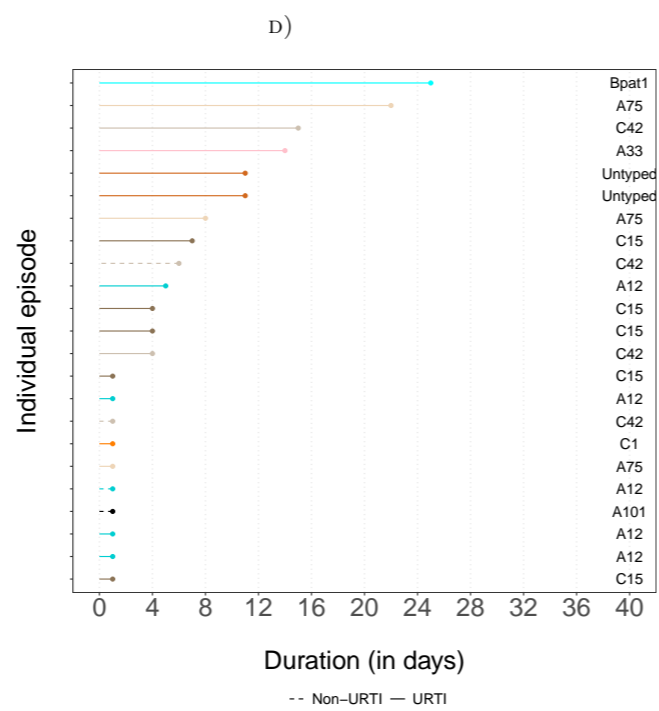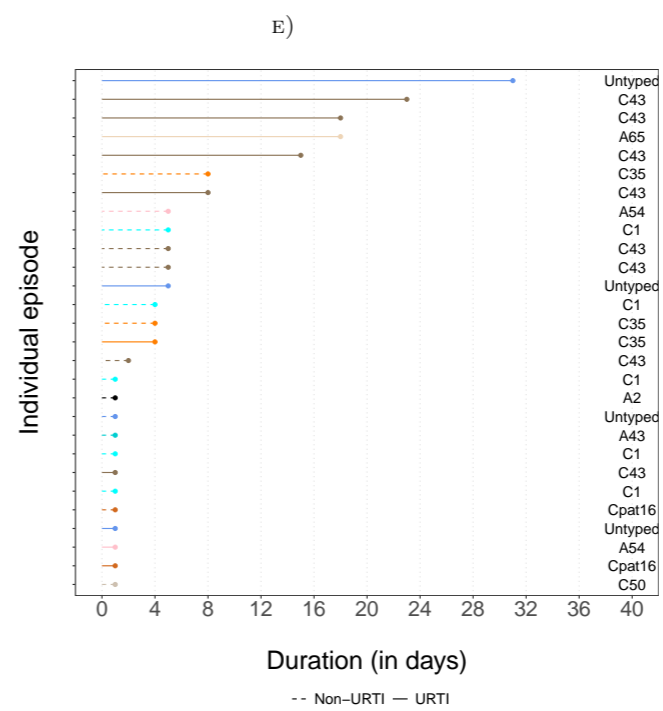

Supplement: Supplementary Figure S3 [file jiy621_suppl_supplementary_figure-s3.pdf]

# HH episode

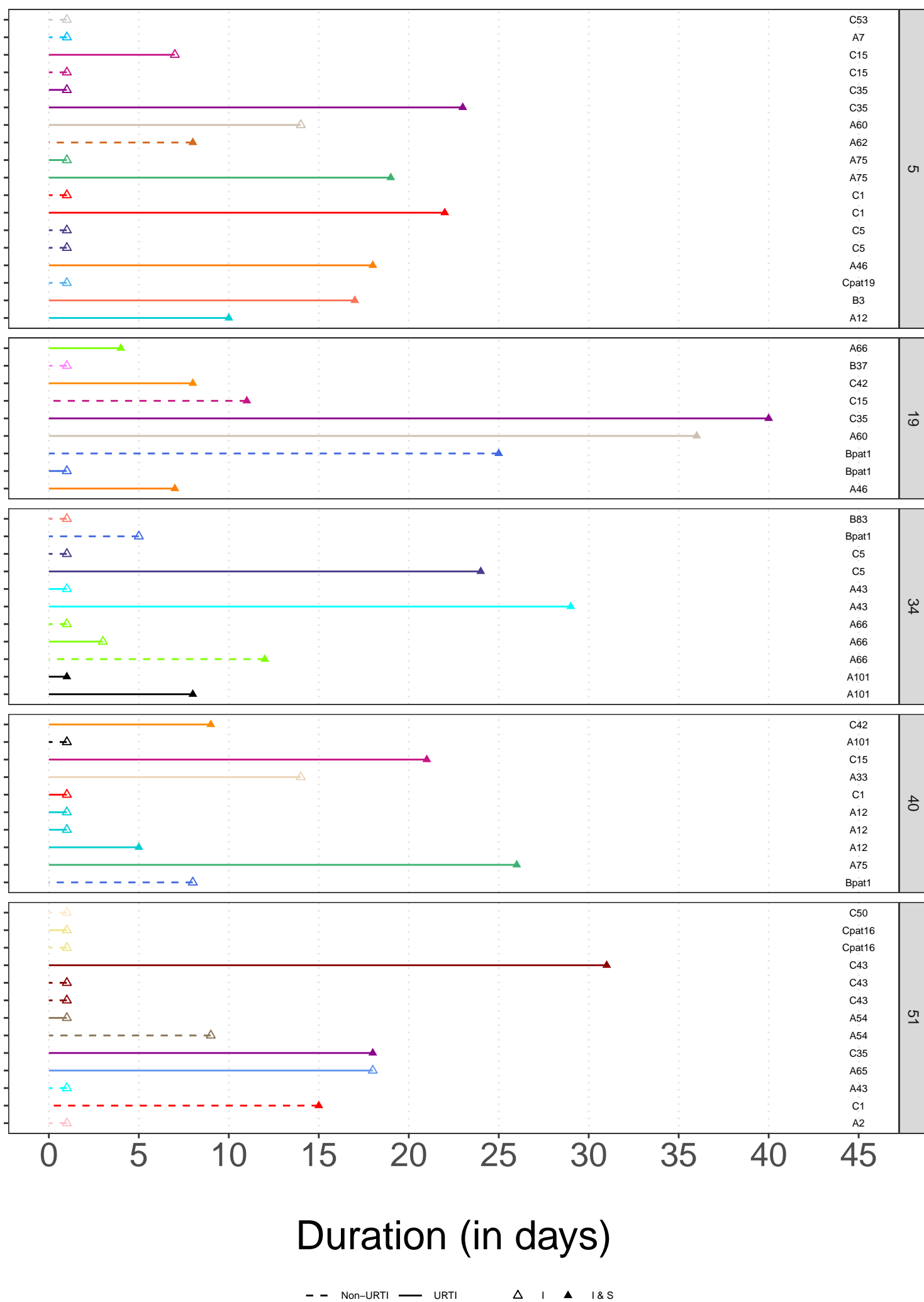

Supplement: Supplementary Figure S4 [file jiy621_suppl_supplementary_figure-s4.pdf]

**A.**

Source

Household  
Inpatient

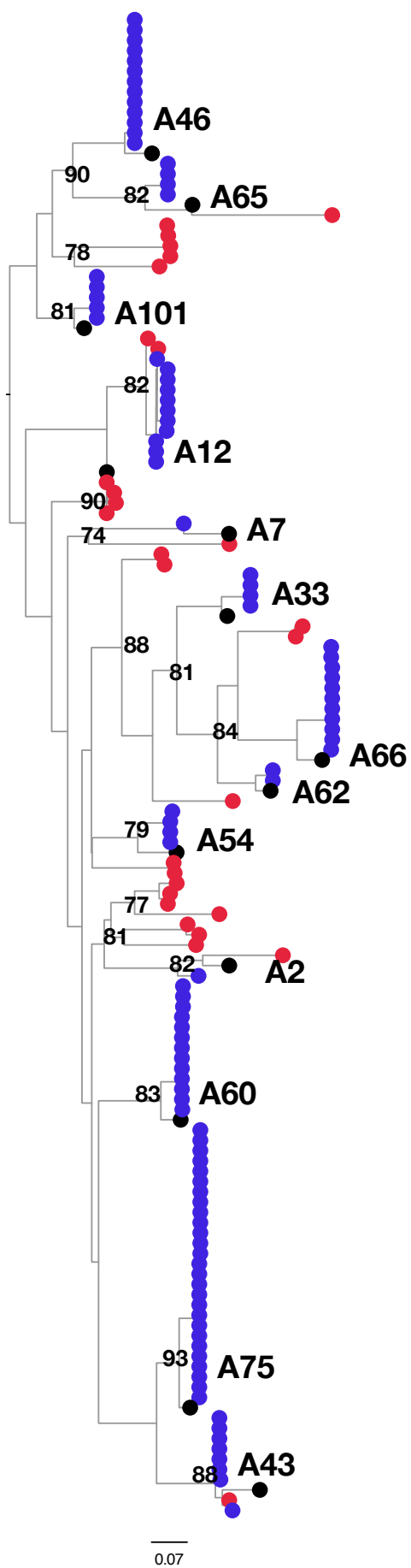**B.**

Source

Household  
Inpatient

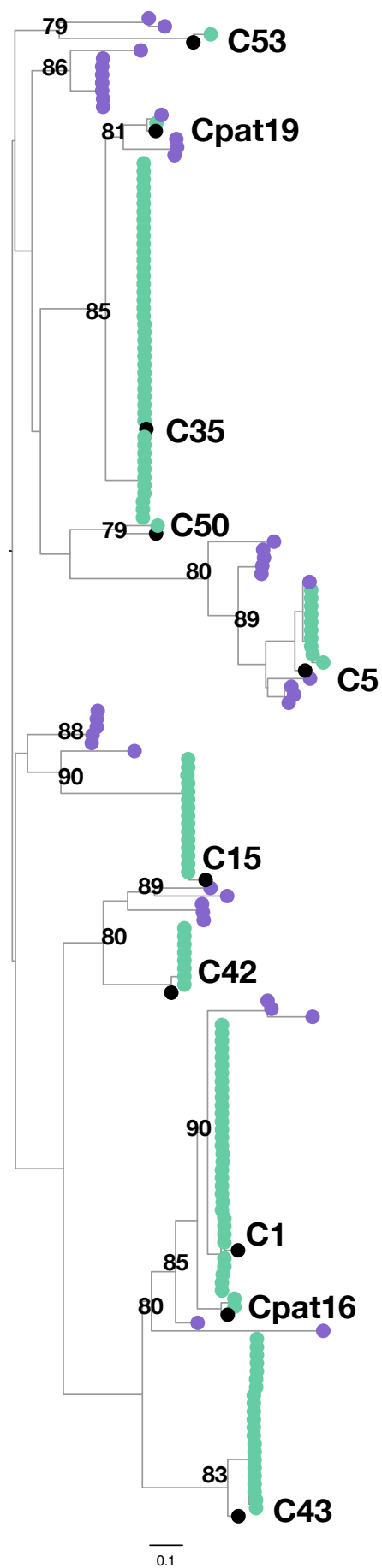

Supplement: Supplementary Figure S5 [file jiy621_suppl_supplementary_figure-s5.pdf]
