# Supplementary material for: An Intensive, Active Surveillance Reveals Continuous Invasion and High Diversity of Rhinovirus in Households
Source: J Infect Dis. 2018 Dec 20;219(7):1049–57. doi: 10.1093/infdis/jiy621 (PMC6420174; doi:10.1093/infdis/jiy621)
Supplement: Supplementary Material [file jiy621_suppl_supplementary_material.docx]

**An intensive, active surveillance reveals continuous invasion and high diversity of rhinovirus in households**

Everlyn Kamau^1*^, Clayton O. Onyango^1,2*#^, Grieven P. Otieno^1^, Patience K. Kiyuka^1^, Charles N. Agoti^1,3^, Graham F. Medley^4^, Patricia A. Cane^5^, D. James Nokes^1,6^, Patrick K. Munywoki^[[1]](#footnote-1),3,**^

*Author Affiliations*

^1^ Epidemiology and Demography Department, Kenya Medical Research Institute (KEMRI) – Wellcome Trust Research Programme, Kilifi, Kenya

^2^ Centers for Disease Control and Prevention, Nairobi, Kenya

^3^ School of Health and Human Sciences, Pwani University, Kilifi, Kenya

^4^ Centre for Mathematical Modelling of Infectious Disease and Department of Global Health and Development, London School of Hygiene and Tropical Medicine, London, United Kingdom

^5^ Public Health England, Salisbury, United Kingdom

^6^ School of Life Sciences and Zeeman Institute for Systems Biology & Infectious Disease Epidemiology Research (SBIDER), University of Warwick, Coventry, United Kingdom

^*^ Joint first authors

^**^*Corresponding Author:*

*Running title:* Rhinovirus transmission in households

***Supplementary Material***

*Table S1: Pairwise nucleotide p-distances between prototype strains and the household sequences.*

| **HRV type** | ***p*-distance** |
| --- | --- |
| **A75** | 0.06-0.063 |
| **A60** | 0.084-0.086 |
| **A12** | 0.076-0.089 |
| **A7** | 0.089 |
| **A46** | 0.068 |
| **A65** | 0.108* |
| **A62** | 0.06 |
| **A66** | 0.108* |
| **A43** | 0.076-0.081 |
| **A33** | 0.065 |
| **A98** | 0.087-0.093 |
| **A2** | 0.106* |
| **A101** | 0.065 |
| **B3** | 0.102* |
| **B37** | 0.097* |
| **Bpat1** | 0.033-0.064 |
| **C1** | 0.039-0.047 |
| **C15** | 0.044-0.047 |
| **C53** | 0.041 |
| **C5** | 0.049-0.083 |
| **Cpat19** | 0.044 |
| **C35** | 0.005-0.01 |
| **C42** | 0.034 |
| **C43** | 0.08-0.088 |
| **Cpat16** | 0.054 |
| **C52** | 0.016 |

* marks types that violated previously proposed assignment thresholds.

***Figure S1:*** *Age-specific prevalence of HRV-A, HRV-B and HRV-C in nasopharyngeal samples collected from symptomatic and asymptomatic individuals.*

***Figure S2:*** *Distribution of human rhinovirus positive samples collected in four other households in coastal Kenya, demonstrated by individual coded by type (colour) and symptom status (filled markers, symptomatic; empty markers, asymptomatic) at time of sample collection. As shown, age increases from the infant at the bottom to the oldest member of the household. Household 5 (A), Household 19 (B), Household 34 (C) and Household 51(D).*

***Figure S3:*** *Plot showing the rhinovirus individual episodes (n=163) in five households from coastal Kenya 2009-10, depicting duration of each episode (x axis), with time=1 representing the first day the first sample for each episode was seen in the individual, ordered by decreasing duration of shedding, colour-coded by type and symptom status (continuous line implies symptomatic, broken line implies asymptomatic episode). (A) Household 5 (B) Household 19 (C) Household 34 (D) Household 40 (E) Household 51.*

***Figure S4:*** *Plot showing the rhinovirus household infection episodes (n=56) in five households from coastal Kenya 2009-10, depicting duration of each household episode (x axis), with time=1 representing the first day the first sample for each episode was seen in the household, ordered by decreasing duration of shedding, colour-coded by type and symptom status (continuous line implies symptomatic, broken line implies asymptomatic episode), ‘I’ refers to episodes with only an index case, while ‘I & S’ refers to those episodes with secondary cases.*

***Figure S5:*** *Human rhinovirus VP4/VP2 phylogenies from coastal Kenya 2009-10, grouping household sequences with contemporaneous Kilifi County Hospital (KCH) sequences: (a) HRV-A, and (b) HRV-C. Tips are coloured based on source of sample, either from the households or KCH (inpatient). Red and Blue tips represent inpatient and household HRV-A samples, respectively, while Purple and Green represent inpatient and household HRV-C samples, respectively. Sequences of prototype strains are coloured in black.*

1. ^#^ Current affiliation: Centers for Disease Control and Prevention, Nairobi, Kenya [↑](#footnote-ref-1)
